# Energy restriction and Roux-en-Y gastric bypass reduce postprandial α-dicarbonyl stress in obese women with type 2 diabetes

**DOI:** 10.1007/s00125-016-4009-1

**Published:** 2016-06-16

**Authors:** Dionne E. Maessen, Nordin M. Hanssen, Mirjam A. Lips, Jean L. Scheijen, Ko Willems van Dijk, Hanno Pijl, Coen D. Stehouwer, Casper G. Schalkwijk

**Affiliations:** 1 Department of Internal Medicine, Laboratory for Metabolism and Vascular Medicine, Maastricht University Medical Center, Peter Debeyelaan 25, P.O. Box 5800, 6202 AZ Maastricht, the Netherlands; 2 Department of Medicine, Division of Endocrinology, Leiden University Medical Center, Leiden, the Netherlands; 3 Department of Human Genetics, Leiden University Medical Center, Leiden, the Netherlands; 4 Einthoven Laboratory for Experimental Vascular Medicine, Leiden, the Netherlands

**Keywords:** Advanced glycation endproducts, α-Dicarbonyls, Obesity, Type 2 diabetes, Weight loss interventions

## Abstract

**Aims/hypothesis:**

Dicarbonyl compounds are formed as byproducts of glycolysis and are key mediators of diabetic complications. However, evidence of postprandial α-dicarbonyl formation in humans is lacking, and interventions to reduce α-dicarbonyls have not yet been investigated. Therefore, we investigated postprandial α-dicarbonyl levels in obese women without and with type 2 diabetes. Furthermore, we evaluated whether a diet very low in energy (very low calorie diet [VLCD]) or Roux-en-Y gastric bypass (RYGB) reduces α-dicarbonyl stress in obese women with type 2 diabetes.

**Methods:**

In lean (*n* = 12) and obese women without (*n* = 27) or with type 2 diabetes (*n* = 27), we measured the α-dicarbonyls, methylglyoxal (MGO), glyoxal (GO) and 3-deoxyglucosone (3-DG), and glucose in fasting and postprandial plasma samples obtained during a mixed meal test. Obese women with type 2 diabetes underwent either a VLCD or RYGB. Three weeks after the intervention, individuals underwent a second mixed meal test.

**Results:**

Obese women with type 2 diabetes had higher fasting and particularly higher postprandial plasma α-dicarbonyl levels, compared with those without diabetes. After three weeks of a VLCD, postprandial α-dicarbonyl levels in diabetic women were significantly reduced (AUC MGO −14%, GO −16%, 3-DG −25%), mainly through reduction of fasting plasma α-dicarbonyls (MGO −13%, GO −13%, 3-DG −33%). Similar results were found after RYGB.

**Conclusions/interpretation:**

This study shows that type 2 diabetes is characterised by increased fasting and postprandial plasma α-dicarbonyl stress, which can be reduced by improving glucose metabolism through a VLCD or RYGB. These data highlight the potential to reduce reactive α-dicarbonyls in obese individuals with type 2 diabetes.

***Trial registration*:**

ClinicalTrials.gov NCT01167959

**Electronic supplementary material:**

The online version of this article (doi:10.1007/s00125-016-4009-1) contains peer-reviewed but unedited supplementary material, which is available to authorised users.

## Introduction

Postprandial glucose excursions are a detrimental factor in diabetic complications [1]. An important mechanism for postprandial glucose peaks contributing to increased risk of diabetic complications may be the formation of α-dicarbonyls, which have been linked to a range of detrimental effects on cellular function [2].

We recently showed that individuals with type 2 diabetes had higher plasma levels of the α-dicarbonyls methylglyoxal (MGO), glyoxal (GO) and 3-deoxyglucosone (3-DG), after a glucose load [3]. These highly reactive α-dicarbonyls are mainly formed as glycolytic intermediates during glucose metabolism and rapidly interact with protein residues [2]. Importantly, α-dicarbonyl stress has been linked to diabetic complications [2]. However, whether type 2 diabetes is associated with higher plasma α-dicarbonyls after a meal is unknown.

Weight loss interventions, particularly energy restriction and bariatric surgery, have been linked to improved glucose metabolism and reduction of diabetic complications [4]. Interestingly, beneficial effects of energy restriction and bariatric surgery on insulin resistance seem to occur rapidly [5]. Thus, it is likely that α-dicarbonyls will be rapidly reduced by these interventions.

Therefore, we investigated whether type 2 diabetes is associated with higher fasting and postprandial plasma α-dicarbonyl levels compared with lean and obese women with normal glucose tolerance (NGT) and whether a diet very low in energy or Roux-en-Y gastric bypass (RYGB) reduces plasma α-dicarbonyl levels in obese women with type 2 diabetes.

## Methods

### Study design and population

The research design of this study has been described previously [5]. In short, we included obese women with NGT (*n* = 27) or type 2 diabetes (*n* = 27) who were eligible for dietary or surgical treatment. Age-matched, healthy women with normal BMI (<25 kg/m^2^; *n* = 12) were included as controls for baseline comparisons. NGT obese individuals underwent either gastric banding (GB, *n* = 11) or RYGB (*n* = 16). Obese individuals with type 2 diabetes either underwent a diet very low in energy (very low calorie diet, VLCD, *n* = 12) or RYGB (*n* = 15). All women were characterised at baseline and obese women were additionally characterised 3 weeks after weight loss intervention. After discontinuing glucose-lowering medication for 48 h and overnight fasting (≥10 h), participants underwent a liquid mixed meal test (MMT) which consisted of 266 ml (1673.6 kJ [400 kcal]) Nutridrink (Nutricia, Zoetermeer, the Netherlands) [5]. The study protocol (clinical trial registration no. NCT01167959) was approved by the medical ethical committee of the Leiden University Medical Center and all participants provided written informed consent. All analyses were performed blind.

### Biochemical measurements

α-Dicarbonyl levels in EDTA plasma samples were measured as described previously [6]. Serum glucose levels and insulin resistance were assessed as described previously [5].

### Calculation of area under the curve

The AUC for α-dicarbonyls and glucose measured during the MMT was calculated using the trapezoidal rule, where fasting levels were subtracted from each individual data point to specify postprandial excursions (incremental AUC, iAUC [nmol/l × min for α-dicarbonyls and mmol/l × min for glucose]).

### Statistical analysis

Baseline levels of α-dicarbonyls and glucose were compared using one-way ANOVA with Bonferroni correction. Two-way repeated measures ANOVA with Bonferroni correction was used to compare the three groups at baseline during the MMT. Adjustment for plasma glucose levels was performed with one-way ANCOVA with Bonferroni correction. Paired two-sided sample *t* tests were used to compare α-dicarbonyl and glucose levels before and after weight loss interventions. Data are expressed as means (SEM), unless otherwise stated. All statistical analyses were performed with IBM SPSS Statistics Software, version 20 (IBM Corporation, Armonk, NY, USA) and a two-sided *p* value of <0.05 was considered statistically significant.

## Results

Baseline characteristics of the study population are reported in Table 1 [5].

**Table 1:** Baseline characteristics of the study population

Characteristic	Lean (*n* = 12)	Obese NGT (*n* = 27)	Obese T2DM (*n* = 27)
Age (years)	49.2 ± 6.2	47.7 ± 6.4	51.0 ± 7.1
Weight (kg)	64.4 ± 7.2	124.3 ± 11.7***	117.2 ± 17.1
BMI (kg/m^2^)	21.7 ± 1.6	43.8 ± 3.2***	42.0 ± 5.5
Waist (cm)	78.0 ± 6.0	122.3 ± 9.2***	123.2 ± 11.0
Fat mass (%)	35.5 ± 2.4	56.3 ± 2.2***	55.7 ± 4.4
HbA_1c_ (mmol/mol)	31.9 ± 2.5	36.1 ± 7.8	49.6 ± 12.0^†††^
HbA_1c_ (%)	5.1 ± 0.2	5.5 ± 0.7	6.7 ± 1.1^†††^
Triacylglycerols (mmol/l)	1.0 ± 0.3	1.4 ± 0.6	1.8 ± 0.7^†^
NEFA (mmol/l)	0.9 ± 0.3	1.0 ± 0.4	1.2 ± 0.3
Total cholesterol (mmol/l)	5.0 ± 0.9	4.6 ± 1.0	4.4 ± 0.8
HDL cholesterol (mmol/l)	1.7 ± 0.3	1.1 ± 0.3***	1.1 ± 0.3
LDL cholesterol (mmol/l)	2.9 ± 0.9	2.9 ± 0.9	2.5 ± 0.6
HOMA-IR	0.3 ± 0.1	3.2 ± 2.3**	5.4 ± 3.6^†^

Data are presented as means ± SD

Differences between the groups were tested using a one-way ANOVA with Bonferroni correction

**p < 0.01 and ***p < 0.001 for lean vs obese NGT individuals.

^†^ *p* < 0.05 and ^†††^ *p* < 0.001 for obese NGT individuals vs obese individuals with type 2 diabetes (T2DM)

### Fasting plasma MGO levels are higher in obese NGT women

**Fig. 1:**
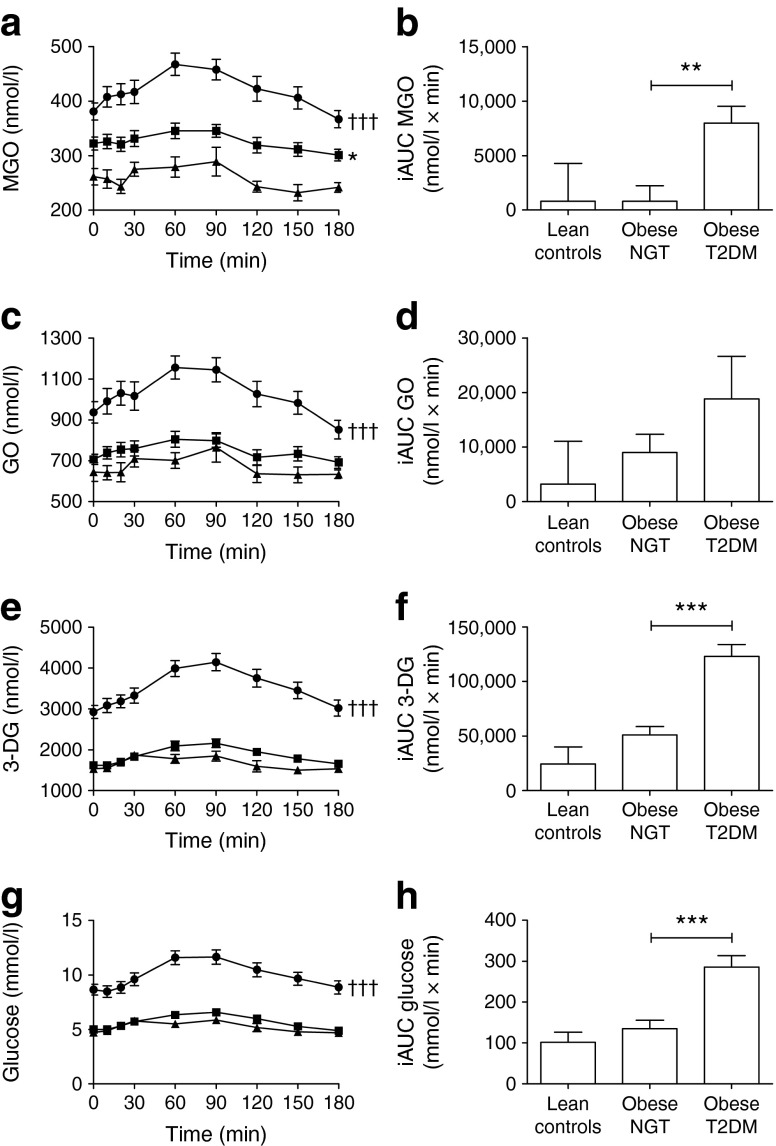
Baseline plasma levels of α-dicarbonyls and glucose before and during a mixed meal test. Plasma levels during the MMT of (**a**) MGO, (**c**) GO, (**e**) 3-DG and (**g**) glucose and iAUC, as calculated from the MMT of (**b**) MGO, (**d**) GO, (**f**) 3-DG and (**h**) glucose. Data are shown as means (SEM). Triangles, lean, *n* = 12; squares, obese NGT, *n* = 27; circles, obese type 2 diabetic individuals, *n* = 27. Differences in postprandial curves during the mixed meal between the groups were tested with repeated-measures two-way ANOVA with Bonferroni correction. Differences in the iAUCs of MGO, GO, 3-DG and glucose between the groups were tested with one-way ANOVA with Bonferroni correction. *p < 0.05, **p < 0.01 and ***p < 0.001 compared with lean individuals and ^†††^ *p* < 0.001 compared with obese NGT individuals

Fasting plasma MGO levels were higher in obese NGT individuals compared with lean individuals (see electronic supplementary material [ESM] Table 1, *p* < 0.05), which remained significant after adjustment for glucose (ESM Table 1). None of the other fasting α-dicarbonyls were increased in obese NGT individuals (ESM Table 1). Furthermore, iAUCs of all plasma α-dicarbonyls and glucose did not differ between lean and obese NGT individuals (Fig. 1b,d,f,h).

### Fasting and postprandial α-dicarbonyl stress is higher in obese women with type 2 diabetes

When we compared obese individuals with NGT and those with type 2 diabetes at baseline, we found significantly higher fasting plasma levels of α-dicarbonyls (ESM Table 1, *p* < 0.01 for all) in those with type 2 diabetes. Particularly, the postprandial iAUCs of MGO, 3-DG and glucose were higher (*p* < 0.01 for all) in obese individuals with type 2 diabetes compared with obese NGT individuals (Fig. 1b,f,h). We observed a similar, yet non-significant difference between obese NGT and type 2 diabetic individuals for the iAUC of GO (Fig. 1d). After adjustment for glucose, all significant differences between obese individuals without and with type 2 diabetes were attenuated (ESM Table 1).

### Fasting plasma MGO levels are reduced by RYGB in obese NGT women within 3 weeks

In obese NGT individuals, we observed reduced fasting MGO levels after RYGB (*p* < 0.05), but no significant reduction in other α-dicarbonyls (ESM Fig. 1). Weight loss through GB in obese NGT individuals did not lead to changes in α-dicarbonyl stress (ESM Fig. 2).

### A VLCD and RYGB reduce fasting and postprandial plasma α-dicarbonyl stress in obese women with type 2 diabetes

**Fig. 2:**
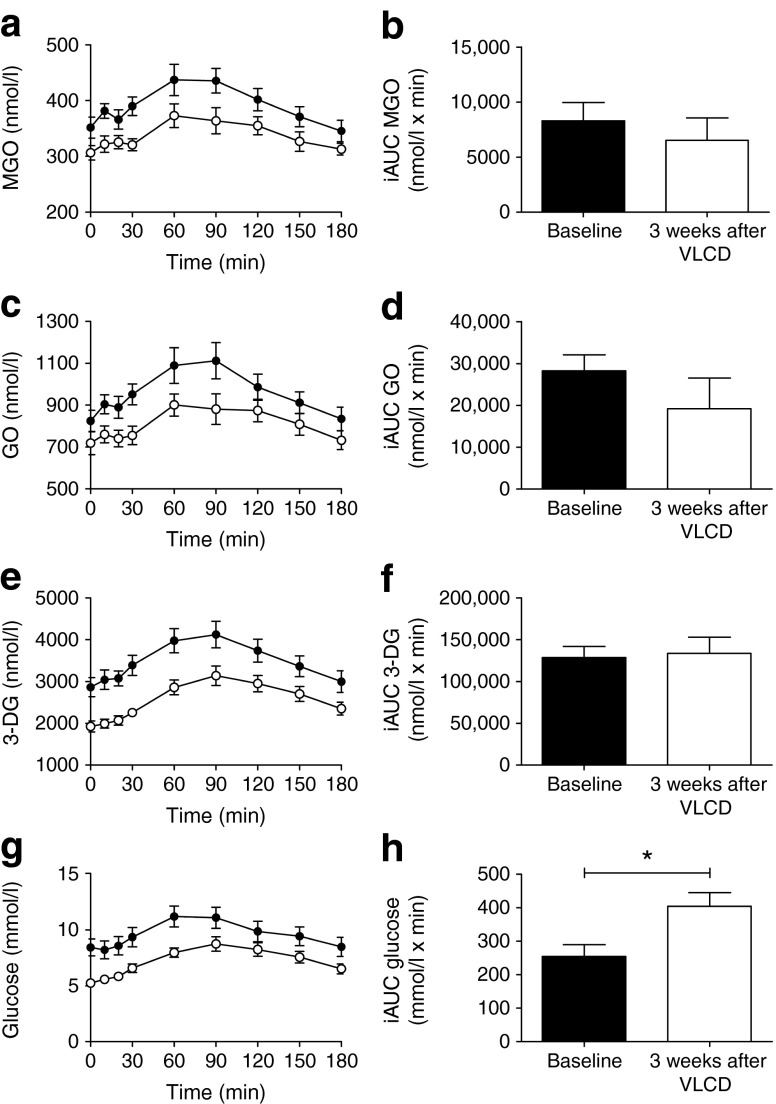
Plasma levels of α-dicarbonyls and glucose in obese individuals with type 2 diabetes after 3 weeks of a VLCD. Plasma levels during the MMT of (**a**) MGO, (**c**) GO, (**e**) 3-DG and (**g**) glucose and iAUC, as calculated from the MMT, of (**b**) MGO, (**d**) GO, (**f**) 3-DG and (**h**) glucose. Data are shown as means (SEM). Black circles, obese type 2 diabetes individuals before the VLCD; open circles, obese type 2 diabetes individuals 3 weeks after the VLCD, *n* = 12. Differences in fasting levels and the iAUCs of MGO, GO, 3-DG and glucose were tested with paired two-sided samples *t* tests. *p < 0.05 compared with baseline

In obese type 2 diabetic individuals, postprandial α-dicarbonyls and glucose levels were decreased after 3 weeks of a VLCD (Fig. 2a,c,e,g). Indeed, the total load of α-dicarbonyls and glucose after a meal, as reflected by the AUC, was substantially decreased by the VLCD (data not shown, *p* < 0.05 for all). This effect was mostly attributable to decreased fasting α-dicarbonyls (MGO and 3-DG, *p* < 0.05 for both), as the postprandial α-dicarbonyl excursions (iAUC) after a meal were similar before and after the VLCD (Fig. 2b,d,f). Fasting plasma GO levels did not change significantly after 3 weeks of the VLCD, but a non-significant (*p* = 0.11) reduction was observed. Comparable findings regarding the effect on postprandial α-dicarbonyl stress were found 3 weeks after RYGB (ESM Fig. 3).

## Discussion

This study shows that obese individuals with type 2 diabetes have higher fasting and particularly higher postprandial plasma α-dicarbonyl levels than individuals without diabetes. Moreover, VLCD and RYGB reduced plasma α-dicarbonyl stress in obese type 2 diabetic individuals, mainly through reduction of fasting plasma α-dicarbonyls.

In obese NGT individuals, we found higher fasting plasma MGO levels compared with lean individuals, but we observed no further postprandial increase. Interestingly, these increased fasting MGO levels were reduced by RYGB. The elevated fasting MGO levels in obese individuals compared with healthy individuals remained significant after adjustment for glucose. Therefore, other pathways may contribute to increased MGO formation in obese NGT individuals. In contrast, fasting and postprandial plasma levels of the other α-dicarbonyls were not affected by the presence of obesity.

Our current findings on increased postprandial α-dicarbonyl stress in type 2 diabetes, together with similar findings in type 1 diabetes [7], are in line with our previous work, in which we demonstrated increased α-dicarbonyl stress in type 2 diabetic individuals after an oral glucose load [3]. Therefore, it is likely that the increased α-dicarbonyl levels after a meal result from increased postprandial glucose levels. Furthermore, the possible role of other substrates for α-dicarbonyl formation, such as reduced lipid oxidation and reactive oxygen species (ROS) cannot be excluded. ROS are known to increase postprandially in type 2 diabetes [8] and, therefore, reduction of ROS by weight loss could also be responsible for lower postprandial MGO stress, but this phenomenon deserves further investigation. Additionally, a VLCD may reduce MGO via induction of nuclear factor (erythroid-derived 2)-like 2 (Nrf2), an inducer of the MGO-detoxifying enzyme glyoxalase-1 [9].

Our current findings are of potential clinical importance, since α-dicarbonyls have been identified as potential key mediators of diabetic complications. Of the three major α-dicarbonyls, MGO is the most potent glycating agent [2]. MGO seems to play a crucial role in the development of diabetic complications, particularly via induction of endothelial dysfunction. Therefore, excursions in MGO at the fasting and postprandial level in type 2 diabetic individuals are a potential target for prevention and treatment of diabetic complications. For example, quenching α-dicarbonyls may be possible with the vitamin B_6_ analogue, pyridoxamine [10]. Furthermore, MGO may be reduced via induction of glyoxalase-1, using inducers of Nrf2 [9].

This is the first study that describes increased postprandial α-dicarbonyl stress in obese type 2 diabetic individuals, and its reduction by weight loss. Future work should investigate whether a VLCD and RYBG achieve health improvements and lower cardiovascular risk through reduced α-dicarbonyl stress. A limitation of this study was that we could not include a control group that remained weight-stable. Nevertheless, we report on two distinct interventions that aimed to induce weight loss, showing comparable results. Another limitation was that we lacked statistical power to investigate mediating factors through which α-dicarbonyl levels were improved by VLCD and RYGB. Additionally, we investigated women only in the current study, but we have no basis to assume that our results would be different in men. Furthermore, our study does not reveal which tissues contribute to the plasma pool of α-dicarbonyls. Although we hypothesise that plasma α-dicarbonyls are derived from insulin-independent cells which are in close contact with blood, such as endothelial cells and erythrocytes, animal studies are needed to fully address this issue.

In conclusion, we demonstrated increased postprandial α-dicarbonyl stress in obese individuals with type 2 diabetes, which can be reduced by a VLCD or RYGB. These data highlight the potential to reduce α-dicarbonyls as a target to prevent or delay development of complications in obese type 2 diabetic individuals.

## Electronic supplementary material

Below is the link to the electronic supplementary material.

ESM Fig. 1 (PDF 193 kb)

ESM Fig. 2 (PDF 118 kb)

ESM Fig. 3 (PDF 116 kb)

ESM Table 1 (PDF 90 kb)

## Abbreviations

3-DG: 3-Deoxyglucosone
GB: Gastric banding
GO: Glyoxal
iAUC: Incremental area under the curve
MGO: Methylglyoxal
MMT: Mixed meal test
NGT: Normal glucose tolerance
Nrf2: Nuclear factor (erythroid-derived 2)-like 2
ROS: Reactive oxygen species
RYGB: Roux-en-Y gastric bypass
VLCD: Very low calorie diet
