# Supplementary material for: Energy restriction and Roux-en-Y gastric bypass reduce postprandial α-dicarbonyl stress in obese women with type 2 diabetes
Source: Diabetologia. 2016 Jun 16;59:2013–7. doi: 10.1007/s00125-016-4009-1 (PMC4969347; doi:10.1007/s00125-016-4009-1)
Supplement: Supplementary file 1 — (PDF 193 kb) [file 125_2016_4009_MOESM1_ESM.pdf]

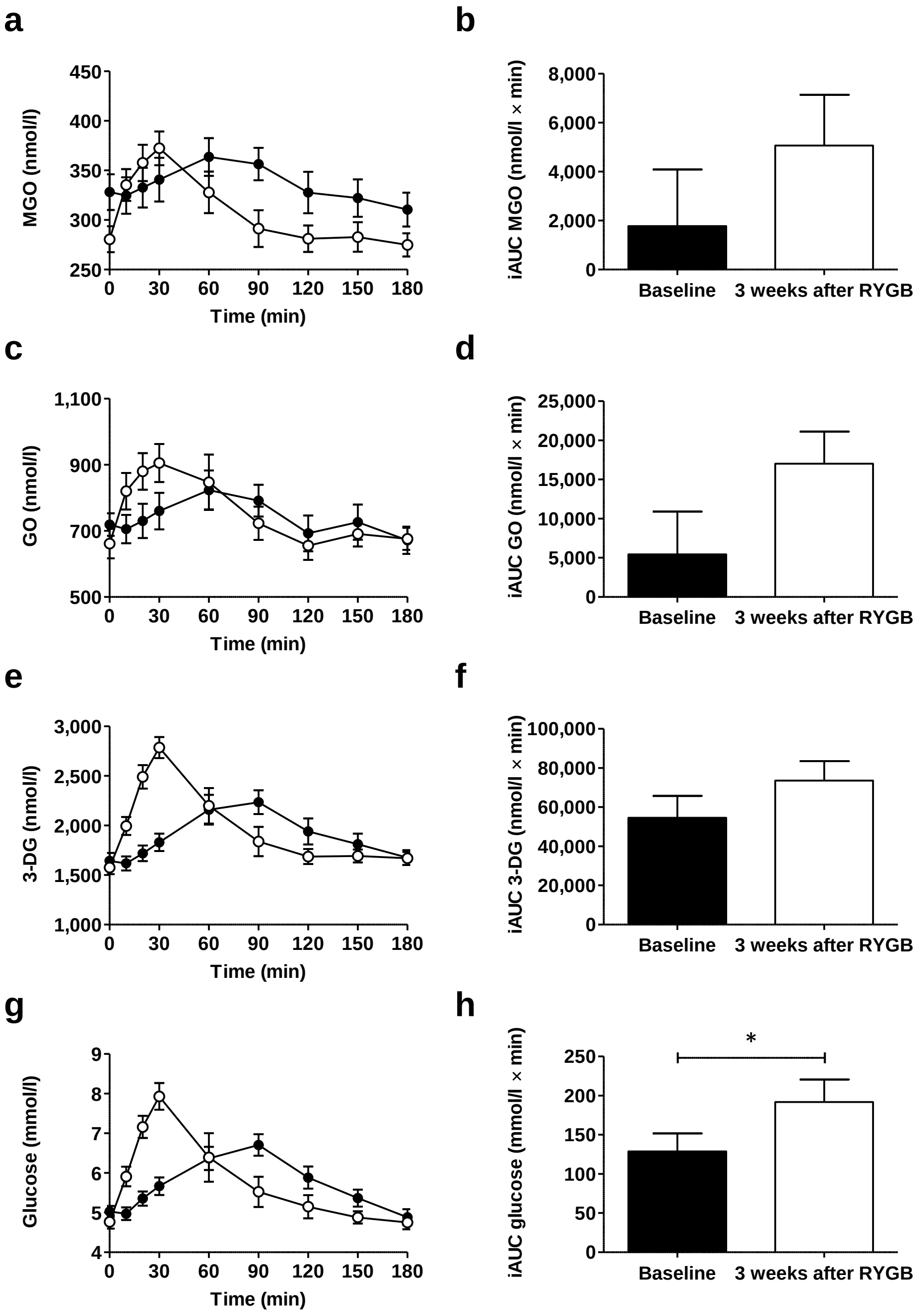

ESM Fig. 1 Plasma levels of α-dicarbonyls and glucose in obese NGT individuals three weeks after Roux-en-Y gastric bypass. Plasma levels during the MMT of a) MGO, c) GO, e) 3-DG and g) glucose. iAUC as calculated from the MMT of b) MGO, d) GO, f) 3-DG and h) glucose. Data are shown as means (SEM). Black circles, obese NGT individuals before RYGB; open circles, obese NGT individuals three weeks after RYGB, *n*=16. Differences in the iAUCs of MGO, GO, 3-DG and glucose were tested with paired two-sided samples *t* tests
