# Supplementary material for: Energy restriction and Roux-en-Y gastric bypass reduce postprandial α-dicarbonyl stress in obese women with type 2 diabetes
Source: Diabetologia. 2016 Jun 16;59:2013–7. doi: 10.1007/s00125-016-4009-1 (PMC4969347; doi:10.1007/s00125-016-4009-1)
Supplement: Supplementary file 4 — (PDF 90 kb) [file 125_2016_4009_MOESM4_ESM.pdf]

ESM Table 1 Plasma levels of fasting and postprandial α-dicarbonyl levels at baseline without and with adjustment for glucose

	Unadjusted means	Adjusted means (adjusted for glucose)
**Fasting MGO (nmol/l)**		
Lean	262 ± 15	290 ± 19
Obese NGT	322 ± 12*	346 ± 14*
Obese T2DM	381 ± 16††	344 ± 16
**Fasting GO (nmol/l)**		
Lean	645 ± 46	729 ± 60
Obese NGT	707 ± 25	777 ± 42
Obese T2DM	937 ± 52†††	829 ± 48
**Fasting 3-DG (nmol/l)**		
Lean	1531 ± 53	2061 ± 81
Obese NGT	1620 ± 61	2063 ± 57
Obese T2DM	2926 ± 159†††	2247 ± 65
**iAUC MGO**		
Lean	795 ± 3487	2057 ± 2718
Obese NGT	823 ± 1424	1542 ± 1673
Obese T2DM	7963 ± 1571††	6759 ± 1795
**iAUC GO**		
Lean	3211 ± 7872	6500 ± 10170
Obese NGT	9006 ± 3368	10925 ± 6270
Obese T2DM	18837 ± 7809	15771 ± 6601
**iAUC 3-DG**		
Lean	24434 ± 15607	39639 ± 15229
Obese NGT	51074 ± 7604	59945 ± 9389
Obese T2DM	123027 ± 10922†††	108853 ± 9884

Lean, *n*=12; obese NGT, *n*=27; obese type 2 diabetes, *n*=27. Data are presented as unadjusted and adjusted means (SEM). Without correction for glucose, differences between the groups were tested with one-way ANOVA with Bonferroni correction. To compare the groups after adjustment for glucose, one-way ANCOVA with Bonferroni correction was used. *$p<0.05$ for lean vs obese NGT individuals. $^{††}p<0.01$ and $^{†††}p<0.001$ for obese NGT vs. obese type 2 diabetes individuals
